# The Association of Resilience with Mental Health in a Large Population-Based Sample (LIFE-Adult-Study)

**DOI:** 10.3390/ijerph192315944

**Published:** 2022-11-29

**Authors:** Elena Caroline Weitzel, Margrit Löbner, Heide Glaesmer, Andreas Hinz, Samira Zeynalova, Sylvia Henger, Christoph Engel, Nigar Reyes, Kerstin Wirkner, Markus Löffler, Steffi G. Riedel-Heller

**Affiliations:** 1 Institute of Social Medicine, Occupational Health and Public Health (ISAP), Medical Faculty, University of Leipzig, Philipp-Rosenthal-Str. 55, 04103 Leipzig, Germany; 2 Department of Medical Psychology and Medical Sociology, Medical Faculty, University of Leipzig, Philipp-Rosenthal-Str. 55, 04103 Leipzig, Germany; 3 Institute of Medical Informatics, Statistics and Epidemiology, University of Leipzig, Härtel-Str. 16–18, 04107 Leipzig, Germany; 4 LIFE—Leipzig Research Centre for Civilization Diseases, University of Leipzig, Philipp-Rosenthal-Str. 27, 04103 Leipzig, Germany

**Keywords:** resilience, depressive symptoms, anxiety symptoms, perceived stress, prevention, general population

## Abstract

Resilience describes a good adaptation to adversity. Strengthening resilience is a promising approach in the prevention of mental health problems. Yet, research on the association of resilience with mental health symptoms in the general population is scarce. The aim of our study is to examine comprehensively the association of resilience with depressive symptoms, anxiety, and perceived stress in a large population-based sample. We analyzed data of n = 3762 participants from the follow-up assessment of the LIFE-Adult-Study, a population-based cohort study in Leipzig. Assessments included resilience (RS-11), depressive symptoms (CES-D), anxiety (GAD-7), and perceived stress (PSQ). The association of resilience with mental health symptoms was examined via multiple linear regression analyses. In our analyses, higher resilience predicted less mental health problems and contributed significantly to the explained variance in mental health outcomes. Women, individuals with previous mental disorders, and those without employment had higher mental health symptoms. Resilience is closely associated with mental health problems in the general population. Vulnerable groups should be targeted with public health measures. Strengthening resilience is a promising approach in the large-scale prevention of mental disorders.

## 1. Introduction

The COVID-19 pandemic has exacerbated mental health problems and has increased the need for effective intervention and prevention programs [1,2]. In order to derive appropriate public mental health measures, a better understanding of the development of mental disorders is necessary [3]. While vulnerability and stress factors have been focused on by previous research, more attention is now falling on protective factors and resources [4]. In this context, the concept of resilience has been developed [5].

Resilience is the “process of adapting well in the face of adversity, trauma, tragedy, threats, or significant sources of stress” [6]. During the COVID-19 pandemic, positive effects of resilience were shown. Lower resilience was found to be significantly associated with worse mental health outcomes during the crisis [7,8,9,10]. High resilience among the elderly was discussed as an underlying factor for relatively stable well-being in the old age population [10,11,12]. Previous studies indicate that resilience tends to be stable or increases over the life course, which could be attributed to life experience and crises successfully overcome [13,14,15].

Resilience integrates various aspects of health-promoting patterns of thinking and behavior [5] and is characterized by an optimistic attitude, adaptive coping styles, and positive emotions [16]. Social support and a large social network are closely associated with resilience [14] illustrating that it is a process emerging dynamically in interaction with the environment [5]. Resilience can be learned and trained [6,17]; it is a promising and universal approach for public mental health measures [18]. In order to evaluate the relevance of the resilience framework for mental health, the relationship between resilience and various mental health symptoms needs to be examined comprehensively.

First, depression is a leading cause of health-related disability and an important public health problem [3,19]. Depressive symptoms are associated with a high level of emotional burden and significant impairments in everyday life, such as impeded community participation [20]. Second, anxiety disorders are the most prevalent group of mental disorders [21]. These often are undetected and at high risk of chronicity [22]. Especially in the elderly, they tend to have a persistent course [23]. Lastly, perceived stress has been identified as a precursor to mental disorders and is therefore a relevant mental health indicator [24]. While it is not a clinical diagnosis, it is a very common phenomenon and has a broad mental and physical health impact [25,26].

The concept and measurement of resilience has been inconsistent in previous research [27]. The dominant view now is of resilience as an interactive and learnable process involving exposure to adversity and positive psychosocial outcomes [5]. In previous studies, resilience was examined partly through self-report [28,29] and partly through psychosocial outcomes such as mental health symptoms [30]. Since the latter neglects positive psychosocial outcomes, and adjustment to adversity may be perceived as good even in the presence of mental health symptoms, resilience is assessed via self-report in this study.

Previous studies suggest the significance of resilience for various mental health symptoms [28,29,31,32,33]. However, these previous studies are limited on the impact of resilience after drastic life events or in specific subpopulations, and neglect the adaptation to adversity in everyday life. Since the relevance of resilience is context-dependent [34], the findings on resilience among people in deep crises cannot be generalized. Scarce studies indicate a positive relation of resilience with mental health symptoms in the general population [13,35], but large-scale, population-based studies including comprehensive sociodemographic, clinical, and social control variables are needed. Knowing more about the relevance of resilience for important mental health symptoms in the general population would provide a starting point in the prevention of mental disorders.

Therefore, our study aims to examine exploratory and comprehensively the association of resilience with different mental health symptoms in a population-based sample of the general population. In order to properly frame the relevance of resilience, we also look at other sociodemographic, clinical, and social correlates of mental health symptoms. We aim to address the following research questions in our study:

1. How is resilience associated with depressive symptoms in the general population?
2. How is resilience associated with anxiety symptoms in the general population?
3. How is resilience associated with perceived stress in the general population?

## 2. Materials and Methods

### 2.1. Sample

**Figure 1:**
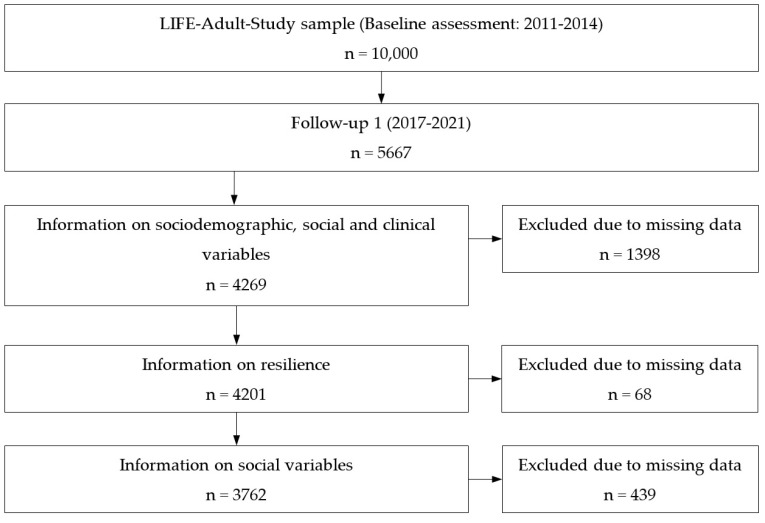
Formation of the study sample.

Data derived from the LIFE-Adult-Study, a population-based cohort study conducted in the city of Leipzig, Germany [36,37]. Resilience was assessed for the first time in the follow-up assessment, so we used data from n = 5667 follow-up participants for the cross-sectional analysis of the relationship of resilience with mental health. The follow-up assessment was conducted between October 2017 and December 2021. As shown in Figure 1, we had to exclude participants due to missing information on sociodemographic, social, and clinical covariates of our analyses (n = 1398). Next, n = 68 participants were excluded due to missing information on resilience. We further excluded n = 439 participants due to missing information in the mental health variables, resulting in a final sample of n = 3762.

Missing data in resilience occurred significantly less often with the female gender (*p* = 0.025), in those with middle (*p* = 0.045) and high education (*p* = 0.003) and more often in participants who were married and living separately (*p* = 0.003), and in those who were divorced (*p* = 0.003). Missing data in mental health variables were less likely in females (*p* = 0.042) and those with middle and high education (*p* = 0.009, *p* = 0.003). Widowhood (*p* = 0.009), part-time work (*p* = 0.027), retirement (*p* < 0.001) and other occupations (*p* = 0.007) were positively associated with missing data in mental health variables.

### 2.2. Assessments

#### 2.2.1. Resilience

The German Resilience Scale-11 (RS-11) was used to measure resilience [4]. The 11-item short version of the Resilience Scale [38] measures resilience with a 7-point Likert scale ranging from 1 = “strongly disagree” to 7 = “strongly agree”. A sum score ranging from 11–77 indicates resilience with higher values representing higher resilience. The 11-item short scale had high internal consistency in our sample (Cronbach’s ɑ = 0.90) and has been validated in the German population [4]. Exemplary items are “I usually manage one way or another”, “I can usually find something to laugh about”, and “I have enough energy to do what I have to do”.

#### 2.2.2. Depressive Symptoms

The German version of the Center for Epidemiologic Studies Depression Scale (CES-D) [39,40] was used to assess depressive symptoms. It is a well established and suitable instrument for measuring depressive symptoms in the general population [40]. The scale consists of 20 items and Cronbach’s ɑ was 0.59 in our sample. The CES-D assesses various depressive symptoms, such as depressive mood, diminished self-esteem, and hopelessness in the past week. Response options are 0 = “rarely or none of the time”, 1 = “some or a little of the time”, 2 = “occasionally or a moderate amount of time”, and 3 = “most or almost all of the time” on a 4-point Likert scale. Answers are summed up to a score ranging from 0 to 60, with higher values indicating greater depressive symptoms. A cut-off of >22 can be used to indicate symptoms of depression [40].

#### 2.2.3. Anxiety Symptoms

Symptoms of General Anxiety Disorder (GAD) were assessed with the German version of the GAD-7 [41,42]. The GAD-7 is a unidimensional scale with 7 items (Cronbach’s ɑ = 0.85 in our sample). It measures anxiety symptoms, such as uncontrollable worries, restlessness, and anxiousness in the past two weeks. The 7 items can be answered on a 4-point Likert scale (0 = “not at all”, 1 = “several days”, 2 = “more than half the days”, and 3 = “nearly every day”). A sum score (range: 0–21) indicates symptoms of GAD with higher values indicating greater symptoms. Spitzer et al. [42] refer to a cut-off of >9 to identify cases of GAD (sensitivity = 89% and specificity = 82%).

#### 2.2.4. Perceived Stress

The Perceived Stress Questionnaire (PSQ) [43] was used to assess perceived stress. Exemplary items are “You feel that too many demands are being made on you”, “You feel tense”, and “You have many worries”. The PSQ consists of 30 items (Cronbach’s ɑ = 0.94 in our sample), which refer to the last 4 weeks and can be answered on a 4-point Likert scale (1 = “almost never”, 2 = “sometimes”, 3 = “often”, or 4 = “usually”). The total score can be transformed into a score ranging from 0 = “lowest possible level of stress” to 1 = “highest possible level of stress” [43]. Cut-off values for high stress can be formed sample-based by adding twice the standard deviation to the mean value [44]. The scale has shown high construct validity in the German general population [44].

#### 2.2.5. Other Measures

Other measures included sociodemographic variables, such as age in years, gender (male or female), marital status (married and living together, married and living separately, single, divorced, or widowed). Information on education and vocational qualifications were classified with regard to the Comparative Analysis of Social Mobility in Industrial Nations (CASMIN) educational classification [45]. Information on occupation included full-time work (≥34 h), part-time work (15–34 h), unemployment, retirement, and other.

Furthermore, clinical variables were assessed. Participants were asked whether they ever had been diagnosed with depression by a physician (“no” “don’t know”, or “yes”) and if they were currently in treatment for depression (“no” or “yes”). They further were asked whether they ever had been diagnosed with an anxiety disorder by a physician (“no”, “don’t know”, or “yes”) and if they were currently being treated for an anxiety disorder (“no” or “yes”). Participants who answered “no” and “don’t know” with regard to the diagnosis were summarized into one group. Additionally, participants in current treatment for depression and/or in current treatment for an anxiety disorder were summarized into one group (“currently in treatment for depression and/or an anxiety disorder”), since these often occur comorbid [21] and we were primarily interested in whether people were currently undergoing any treatment.

Further, social support was assessed with the ENRICHD Social Support Inventory (ESSI) [46,47]. This 5 item instrument consists of questions like “Is there someone available to you whom you can count on to listen to you when you need to talk?”, “Is there someone available to give you good advice about a problem?”, and “Is there someone available to you who shows you love and affection?” with the response options ranging from 1 = “none of the time” to 5 = “all the time” on a 5-point Likert scale. A sum score ranging from 5 to 25 indicates social support, with higher values indicating higher social support.

The short version of the Lubben Social Network Scale (LSNS-6) [48] was used to assess the social network size. This 6-item instrument contains questions on the availability of social contact with family members and friends like “How many relatives do you see or hear from at least once a month?” and “How many friends do you feel close to such that you could call on them for help?”. All items can be answered on a 5-point Likert scale (0 = “none”, 1 = “one”, 2 = “two”, 3 = “three or four”, 4 = “five thru eight”, and 5 = “nine or more”). Answers are summed up to a score ranging from 0–30 with higher values indicating a larger social network.

### 2.3. Statistical Analyses

Data were weighted by age and gender according to German census data to ensure representativeness of the study sample for the German general population. We used t-tests to examine whether mean resilience differed depending on the presence of depressive symptoms, symptoms of GAD, or high stress. The associations of resilience and mental health variables were examined using multiple linear regression analyses. Depressive symptoms, anxiety symptoms, and perceived stress were included as outcome variables; resilience was included as independent variable. Further, the following covariates were added: age group (“18–39 years”, “40–59 years”, or “≥60 years”), gender (“male or “female”), marital status (“married and living together”, “married and living separately”, “single”, “divorced”, or “widowed”), education (“low”, “middle”, or “high”), occupation (“full-time (≥34 h)”, “part-time (15–34 h)”, “unemployed”, “retirement”, or “other”), ever diagnosed with an anxiety disorder (no/don’t know, or yes), ever diagnosed with depression (“no/don’t know”, or “yes”), currently in treatment due to anxiety disorder and/or depression (“no” or “yes”), social support, and social network. For each categorical variable, the first option listed was the reference category.

The association of resilience with mental health outcomes was examined via the significance of resilience as a predictor in the regression model. Further, we conducted each regression analyses with and without resilience as a predictor variable to gain insight in the relevance of resilience in the regression of the outcome variable.

We performed the statistical analyses with R [49], Rstudio [50] and the additional packages survey [51], dplyr [52], and psych [53]. We defined statistical significance with *p* < 0.05.

## 3. Results

### 3.1. Sample Characteristics

Table 1 presents the characteristics of the study sample. The weighted mean age was 53.61 years (*SD* = 16.66) and 53.5% were female (n = 2012). Half of the study participants were married and living with their spouse (52.8%, n = 2405) and 30.1% were single (n = 551). The majority had middle education (57.0%, n = 2080) or high education (38.3%, n = 1503). About half of the sample were working full-time (≥34 h; 52.6%, n = 1520) and about a quarter were retired (27.6%, n = 1662). It was shown that 4.1% (n = 157) said that they had been diagnosed with an anxiety disorder by a physician, and twice as many (8.4%, n = 321) had been diagnosed with depression. Furthermore, 5.0% (n = 189) were currently undergoing treatment due to an anxiety disorder and/or depression.

Descriptives and correlations of resilience and mental health variables can be found in Table S1 in Supplementary Materials. The means of resilience according to symptoms of depression, symptoms of GAD, and perceived stress are presented in Table 2. Resilience was significantly lower in those with symptoms of depression (t(3760) = −8.873, *p* < 0.001), in those with symptoms of GAD (t(3760) = −8.946, *p* < 0.001), and in those with high perceived stress (t(3760) = −8.342, *p* < 0.001).

### 3.2. Results of the Regression Analyses

#### 3.2.1. Resilience and Depressive Symptoms

Table 3 presents the results of the linear regression analyses. Higher resilience significantly predicted lower depressive symptoms (β = −0.136, *p* < 0.001). The proportion of explained variance in depression increased significantly when resilience was added to the model (R^2^ = 0.179 vs. 0.162, *p* < 0.001). In the regression of depressive symptoms, age group, gender, marital status, occupation, having ever been diagnosed with an anxiety disorder, having ever been diagnosed with depression, and social support were significant covariates. Depressive symptoms were lower in participants aged ≥ 65 years compared to those aged 18–39 years (β = −0.198, *p* = 0.018). Depressive symptoms were higher when participants were married and living separately compared to those married and living together (β = 0.269, *p* = 0.034). In addition, depressive symptoms were higher in unemployed participants (β = 0.463, *p* = 0.017) and in those with other occupation status (β = 0.197, *p* = 0.049) in reference to full-time working participants. Further, depressive symptoms were higher in participants who reported the diagnosis of an anxiety disorder (β = 0.585, *p* < 0.001) and depression (β = 0.534, *p* < 0.001). Higher social support was associated with lower depressive symptoms (β = −0.125, *p* < 0.001).

#### 3.2.2. Resilience and Anxiety Symptoms

Higher resilience was associated with lower anxiety symptoms (β = −0.295, *p* < 0.001). The amount of explained variance in anxiety symptoms increased significantly when resilience was added to the model (R^2^ = 0.279 vs. 0.206, *p* < 0.001). Significant covariates in the regression of anxiety symptoms were gender, occupation, having been diagnosed with an anxiety disorder, having been diagnosed with depression, social support, and social network. Female study participants were more likely to have higher anxiety symptoms (β = 0.356, *p* < 0.001). Unemployed participants showed significantly higher anxiety symptoms (β = 0.340, *p* = 0.014), and retired participants showed significantly lower anxiety symptoms (β = −0.124, *p* = 0.031) compared to those working full-time. The diagnosis of an anxiety disorder (β = 0.867, *p* < 0.001) or depression (β = 0.452, *p* < 0.001) in the past was associated with higher anxiety symptoms. Higher social support (β = −0.128, *p* < 0.001) and a larger social network (β = −0.061, *p* = 0.016) predicted lower anxiety symptoms.

#### 3.2.3. Resilience and Perceived Stress

Higher resilience significantly predicted lower perceived stress (β = −0.370, *p* < 0.001). The inclusion of resilience substantially increased the amount of explained variance in the regression of perceived stress (R^2^ = 0.353 vs. 0.243, *p* < 0.001). In the regression of perceived stress, age group, gender, occupation, having been diagnosed with an anxiety disorder, having been diagnosed with depression, social support, and social network were significant covariates. Participants aged 65 years and older were likely to have lower perceived stress than those aged 18–39 years (β = −0.061, *p* = 0.001). Female gender was associated with higher perceived stress (β = 0.294, *p* < 0.001). Part-time work (β = −0.221, *p* = 0.005) and retirement (β = −0.429, *p* < 0.001) predicted lower perceived stress than working full-time. Having been diagnosed with an anxiety disorder was associated with higher perceived stress (β = 0.364, *p* = 0.003), as well as having been diagnosed with depression (β = −0.362, *p* < 0.001). Higher social support (β = −0.171, *p* < 0.001) and a larger social network (β = −0. 059, *p* = 0.016) were associated with lower perceived stress.

## 4. Discussion

In this study, we examined the association of resilience with mental health variables in the general population. We found that resilience was lower in those with symptoms of depression, GAD, and with higher perceived stress, respectively. Resilience was a significant predictor for depressive symptoms, anxiety, and perceived stress in the large population-based LIFE-Adult sample. Higher resilience manifests itself in a lower mental health burden with regard to various different symptoms. The results extend previous findings from at-risk groups and subpopulations to the population as a whole and highlight the high relevance of the resilience framework for mental health.

With regard to the association of resilience with depressive symptoms, previous research found a significant association in women [35]. Other studies revealed an association of resilience with depressive symptoms in age-specific subgroups [30,33] and in individuals with chronic health conditions [28,31,32]. With our investigation, we extended the previous research by identifying a significant role of resilience in predicting depressive symptoms in the general population. A positive attitude towards one’s own ability to withstand and cope with crises could counteract the vulnerability to depressive patterns of thinking and behavior. Further longitudinal studies should examine how resilience affects the incidence of depressive symptoms in the long term.

With regard to the association of resilience with anxiety symptoms, previous research found that higher resilience was linked to lower anxiety in at-risk groups such as cancer patients [28,31]. Correspondingly, in our sample of the general population higher resilience was associated with lower anxiety. Our results illustrate that an optimistic attitude towards coping with crises is accompanied by less burden from worry and anxiety, and extend the previous research by confirming the transferability to the general population. As with depressive symptoms, long-term studies should further investigate the nature of the association between resilience and anxiety.

Further, we found in our study that higher resilience was associated with lower perceived stress. Here, the proportion of explained variance in perceived stress increased most substantially with the inclusion of resilience in the model (24.3% vs. 35.3%). Perceived stress indicates an overload and an insufficient ability to cope with demands in everyday life. In contrast, resilience describes a successful adaptation to adversity. Accordingly, our results map well the inverse nature of these constructs. Higher resilience could possibly reflect better resources for dealing with stressors. Future studies should address the relationship of resilience and stress over time to draw causal conclusions.

In our analyses, we considered a variety of sociodemographic and social covariates to rule out confounding influences. The findings are largely in line with those of other studies on mental health correlates. We confirmed that women were more burdened with regard to all included mental health variables [26,42,54,55]. In line with previous research, being 60 years and older was associated with less depressive symptoms [56] and less perceived stress [55]. Whereas, in concordance with Lenze and Wetherell [23], our results suggest the stability of anxiety symptoms across age.

We further found that depressive symptoms were higher among the married who did not live with their partner. This might indicate that they are currently going through separation, which can be accompanied by acute psychological distress and a depressive mood [57]. In contrast to previous studies [55], neither being single nor divorced or widowed was associated with psychological distress in our analysis, which may be due to a underlying mediating effect of social variables included in the statistical model. Thus, it is possible that marital status influences the availability of social support, which in turn is closely related to psychological wellbeing.

Interestingly, we did not find any association of education with mental health variables. In previous studies, education as the basis for socioeconomic status has been associated with mental health variables [55,58]. This difference might be explained by the inclusion of occupation and social support as a control variable. The latter has been identified as a mediating mechanism in the impact of socioeconomic status on mental health [59].

In addition, in our sample, the association of occupation with mental health was complex. On the one hand, we confirmed previous findings on the association of unemployment with depressive symptoms and anxiety, which could be due to the fact that lower financial resources in unemployment can be a cause for worry and concern about the future [60,61,62]. On the other hand, we found that a higher workload was accompanied by higher perceived stress, and retirement by lower anxiety and stress, which suggests work-related triggers of worries and concern. The findings demonstrate the importance of multidisciplinary approaches with regard to mental health, which should necessarily include vocational reintegration measures and a diminution of occupational overload.

With regard to clinical covariates, people who had been diagnosed with an anxiety disorder or depression were consistently more burdened. We also demonstrated that the presence of symptoms of depression or GAD is associated with lower resilience, indicating higher vulnerability. Particularly in view of the pandemic and the cutback of mental health services, people with mental disorders are at risk of falling off the grid. So far, studies show no significant deterioration in the wellbeing of people with a mental disorder [63], but given the already high treatment gaps [64] and the rising mental health burden [2], this could change in the long-term. Strengthening resilience can be a useful approach even when mental health symptoms are already present. This is clearly illustrated by Yu et al. [65], who found that better resilience in those with a pre-existing diagnosis still had relevant positive correlates such as a lower suicidality [65]. Starting points for strengthening resilience in people with mental illnesses could be the integration of low-threshold peer support in routine care and the promotion of organized self-help, because this involves social support and enables the sharing of experiences of resilient recovery. The focus on resilience factors such as personal resources and coping mechanisms might increase the feeling of self-efficacy and enhance the empowerment of subjects with a mental health diagnose.

In line with previous research, our results reveal a high relevance of social support and inclusion for mental health [66,67,68]. Initial studies indicate that resilience mediates the association between social support and mental health in at-risk populations [28,68,69]. Thus, it is conceivable that in the general population, too, a high availability of social resources results in higher resilience, which in turn has a positive effect on mental health. Future studies should examine whether studies suggesting a mediation effect of resilience can be transferred to the general population.

Our results suggest that resilience is a meaningful correlate of mental health, along with other sociodemographic, social, and clinical covariates. The latter could be used to identify at-risk groups that could particularly benefit from resilience interventions. In this context, another interesting question for future studies is whether the link between resilience and mental health is the same for all groups or whether it is moderated by other factors. Previous studies suggest that resilience tends to be stable across age [13,14,15], whereas younger participants were more burdened by mental health symptoms in our study. This could point out a lower relevance of resilience at younger age, which should be investigated in future studies.

Mental health symptoms can be an appropriate and evolutionary useful reaction to aversive conditions, as they can signal a need to others and regulate disengagement [69]. In this context, resilience could contribute to the explanation of interindividual differences in response to adversity. Resilience could take on a counterbalancing function and indicate the availability of resources such as life experience in this context.

In order to derive and develop resilience strengthening interventions, it is necessary to find out how exactly resilience manifests in cognition and behavior and how it depends on external stressors. This would reveal perspectives and behaviors that people with difficulties in resilient adaption might try to adopt in order to cope better. At the population level, it is important to address factors, which influence mental health and resilience. From a public health perspective, risk factors such as occupational overload or unemployment should be reduced where possible, and protective factors such as social inclusion should be strengthened. In this context, community-based interventions have a large potential and could foster neighborhood integration by offering events and meeting places. In addition, peer support in routine mental healthcare could be more widely encouraged and implemented.

### Strengths and Limitations

We examined the association of resilience and mental health variables in a large sample of the German general population with comprehensive statistical control. Our results, therefore, have high generalizability. Nevertheless, our investigation has some limitations. As information on resilience was only available for the follow-up assessment of the LIFE-Adult-participants, our analyses were limited to cross-sectional analyses. Consequently, as in any cross-sectional analysis, no causal relationship can be inferred from our results. Furthermore, our analyses on the association of resilience with mental health symptoms refer to self-reported data, which are highly correlated. Further long-term studies including clinical diagnoses are needed to shed more light on the relevance of resilience for the incidence of mental disorders.

## 5. Conclusions

Taken together, our results indicate that strengthening resilience is a promising measure to improve a wide range of mental health symptoms. Strengthening resilience corresponds to a universal prevention approach that addresses the often comorbid occurrence of different mental health symptoms [21]. Given that women, individuals with previous mental disorders, and those without employment were particularly burdened in our analyses, these groups should be a higher priority for prevention and intervention programs.

## Figures and Tables

**Table 1:** Sample characteristics.

Variable	Total n = 3762		Women n = 2012		Men n = 1750		*p*
Sociodemographic variables							
Age, *M* (*SD*)	53.61	(16.66)	54.42	(16.83)	52.77	(16.44)	0.190
Age group, n (%)							0.377
18–39 years	149	(25.4)	74	(24.2)	75	(26.6)	
40–59 years	1477	(37.5)	831	(36.7)	646	(38.4)	
≥60 years	2136	(37.1)	1107	(39.1)	1029	(35.1)	
Marital status, n (%)							<0.001
Married (living together)	2405	(52.8)	1170	(49.3)	1235	(56.4)	
Married (living separately)	76	(2.1)	45	(2.1)	31	(2.1)	
Single	551	(30.1)	284	(27.6)	267	(32.6)	
Divorced	455	(9.2)	296	(11.4)	159	(6.9)	
Widowed	275	(5.9)	217	(9.6)	58	(2.1)	
Education, n (%)							<0.001
Low	179	(4.7)	105	(6.8)	74	(2.5)	
Middle	2080	(57.0)	1208	(57.2)	872	(56.8)	
High	1503	(38.3)	699	(36.1)	804	(40.7)	
Occupation, n (%)							<0.001
Full-time (≥34 h)	1520	(52.6)	734	(43.7)	786	(61.9)	
Part-time (15–34 h)	323	(10.6)	273	(16.8)	50	(4.2)	
Unemployed	69	(2.6)	35	(2.7)	34	(2.5)	
Retirement	1662	(27.6)	859	(30.1)	803	(25.0)	
Other	188	(6.5)	111	(6.6)	77	(6.3)	
Clinical variables							
Ever diagnosed with anxiety disorder, n (%)	157	(4.1)	101	(5.0)	56	(3.2)	0.047
Ever diagnosed with depression, n (%)	321	(8.4)	217	(10.6)	104	(6.1)	<0.001
In treatment due to anxiety disorder and/or depression, n (%)	189	(5.0)	120	(6.4)	69	(3.5)	0.004
Social variables							
Social support, *M* (*SD*)	22.50	(3.34)	22.62	(3.31)	22.37	(3.37)	0.040
Social network, *M* (*SD*)	18.04	(5.32)	18.18	(5.19)	17.90	(5.45)	0.724

Notes. *M*, *SD*, and % are weighted by age and gender according to census data, n are unweighted count. Social support and social network were measured with the ENRICHD Social Support Inventory (ESSI), and the Lubben Social Network Scale (LSNS).

**Table 2:** Resilience according to symptoms of depression, general anxiety disorder, and perceived stress.

			**Resilience**		
	**n**	**(%)**	***M***	***SD***	***p***
Total	3762		60.50	10.43	
Symptoms of depression					<0.001
yes	167	(4.0)	48.95	12.67	
no	3595	(96.0)	60.98	10.05	
Symptoms of general anxiety disorder					<0.001
yes	208	(5.8)	50.97	11.43	
no	3554	(94.2)	61.10	10.07	
Perceived stress					<0.001
high	153	(5.0)	49.45	11.44	
low to moderate	3609	(95.0)	61.08	10.05	

Notes. *M*, *SD*, and % are weighted by age and gender according to census data, n are unweighted count. Resilience, depressive symptoms, anxiety symptoms, and perceived stress were assessed with the 11-item Resilience Scale RS-11, the Center for Epidemiologic Studies Depression Scale (CES-D, cut-off: CES-D > 22), the General Anxiety Disorder Scale-7 (GAD-7, cut-off: GAD-7 > 9), and the Perceived Stress Questionnaire (PSQ, cut-off: PSQ > *M* + 2*SD* = 0.56).

**Table 3:** Results of the multiple linear regression analyses ^1^.

Predictor Variables	Depressive Symptoms				Anxiety Symptoms				Perceived Stress			
	β	95% CI Lower Bound	95% CI Upper Bound	*p*	β	95% CI Lower Bound	95% CI Upper Bound	*p*	β	95% CI Lower Bound	95% CI Upper Bound	*p*
***Sociodemographic variables***												
Age group, ref. 18–39 years												
40–59 years	−0.129	−0.367	0.010	0.069	−0.035	−0.190	0.121	0.662	−0.061	−0.217	0.095	0.441
≥60 years	−0.198	−0.363	−0.034	**0.018**	−0.071	−0.239	0.097	0.406	−0.311	−0.489	−0.133	**0.001**
Gender, ref. male												
Female	0.324	0.241	0.406	**<0.001**	0.356	0.265	0.446	**<0.001**	0.294	0.206	0.383	**<0.001**
Marital status, ref. married and Living together												
Married and living seperatly	0.269	0.021	0.518	**0.034**	0.012	−0.268	0.292	0.931	0.065	−0.223	0.354	0.656
Single	0.069	−0.049	0.187	0.253	0.023	−0.103	0.148	0.724	−0.050	−0.181	0.082	0.459
Divorced	0.045	−0.080	0.171	0.479	−0.032	−0.140	0.076	0.565	−0.094	−0.211	0.023	0.116
Widowed	0.103	−0.074	0.280	0.254	0.055	−0.091	0.201	0.461	0.011	−0.137	0.159	0.884
Education, ref. low												
Middle	−0.108	−0.302	0.086	0.277	−0.039	−0.222	0.144	0.674	−0.089	−0.299	0.121	0.404
High	−0.072	−0.273	0.129	0.482	0.036	−0.153	0.225	0.709	0.085	−0.129	0.299	0.434
Occupation, ref. full-time (≥34 h)												
Part-time (15–34 h)	−0.123	−0.286	0.040	0.140	−0.110	−0.260	0.040	0.150	−0.221	−0.376	−0.067	**0.005**
Unemployed	0.463	0.082	0.845	**0.017**	0.340	0.070	0.611	**0.014**	0.077	−0.225	0.376	0.618
Retirement	0.006	−0.115	0.126	0.927	−0.124	−0.238	−0.011	**0.031**	−0.429	−0.541	−0.318	**<0.001**
Other	0.197	0.001	0.393	**0.049**	0.038	−0.138	0.215	0.668	−0.018	−0.262	0.226	0.882
***Clinical variables***												
Ever diagnosed with anxiety disorder	0.585	0.291	0.880	**<0.001**	0.867	0.525	1.210	**<0.001**	0.364	0.120	0.608	**0.003**
Ever diagnosed with depression	0.534	0.285	0.784	**<0.001**	0.452	0.255	0.648	**<0.001**	0.362	0.170	0.555	**<0.001**
In treatment due to anxiety disorder and/or depression	0.073	−0.262	0.409	0.668	0.003	−0.335	0.341	0.986	0.161	−0.142	0.463	0.299
***Social variables***												
Social support	−0.125	−0.182	−0.067	**<0.001**	−0.128	−0.183	−0.072	**<0.001**	−0.171	−0.222	−0.120	**<0.001**
Social network	0.044	−0.002	0.090	0.058	−0.061	−0.110	−0.011	**0.016**	−0.059	−0.107	−0.011	**0.016**
***Resilience***	−0.136	−0.184	−0.088	**<0.001**	−0.295	−0.355	−0.235	**<0.001**	−0.370	−0.433	−0.306	**<0.001**
***Model variables***												
R^2^	0.179				0.279				0.353			

Notes. Results are weighted by age and gender according to census data. Depressive symptoms, anxiety symptoms, perceived stress, social support, social network, and resilience were measured with the the Center for Epidemiologic Studies Depression Scale (CES-D), the General Anxiety Disorder Scale-7 (GAD-7), the Perceived Stress Questionnaire (PSQ), the ENRICHD Social Support Inventory (ESSI), the Lubben Social Network Scale (LSNS), and the 11-item Resilience Scale (RS-11). Bold *p*-values indicate significance. ^1^ Multiple linear regression analyses were performed with depressive symptoms, anxiety symptoms, and perceived stress as outcome and other variables as predictor variables.

## Data Availability

The dataset analysed during the current study is available from the corresponding author upon reasonable request.

## Supplementary Materials

The following supporting information can be downloaded at: https://www.mdpi.com/article/10.3390/ijerph192315944/s1, Table S1: Descriptives and correlations of resilience and mental health symptoms in the study sample.
