# Supplementary material for: The Association of Resilience with Mental Health in a Large Population-Based Sample (LIFE-Adult-Study)
Source: Int J Environ Res Public Health. 2022 Nov 29;19(23):15944. doi: 10.3390/ijerph192315944 (PMC9740913; doi:10.3390/ijerph192315944)
Supplement: Supplementary file 1 [file ijerph-19-15944-s001.zip › ijerph-2021236-supplementary.pdf]

Table S1. Descriptives and correlations of resilience and mental health symptoms in the study sample.

	M	***SD***	**Resilience** ***r***	**Depressive Symptoms** ***r***	**Anxiety Symptoms** ***r***
Resilience	60.50	10.43	1		
Depressive symptoms	13.25	4.77	−0.227**	1	
Anxiety symptoms	3.59	3.36	−0.393**	0.551**	1
Perceived stress	0.26	0.15	−0.451**	0.448**	0.734**

Notes. *M*, *SD* and *r* are weighted by age and gender according to census data. Resilience, depressive symptoms, anxiety symptoms, and perceived stress were assessed with the 11-item Resilience Scale RS-11, the Center for Epidemiologic Studies Depression Scale (CES-D, cut-off: CES-D > 22), the General Anxiety Disorder Scale-7 (GAD-7, cut-off: GAD-7 > 9), and the Perceived Stress Questionnaire (PSQ, cut-off: PSQ > M + 2SD = 0.56).

** $p < 0.001$.
